# Adverse perinatal outcomes in gestational diabetes mellitus with and without SARS-CoV-2 infection during pregnancy: results from two nationwide registries in Germany

**DOI:** 10.1136/bmjdrc-2023-003724

**Published:** 2024-01-25

**Authors:** Tatjana P Liedtke, Katharina S Weber, Heinke Adamczewski, Dietmar Weber, Babett Ramsauer, Ute M Schaefer-Graf, Tanja Groten, Eike A Strathmann, Wolfgang Lieb, Mario Rüdiger, Ulrich Pecks, Helmut J Kleinwechter

**Affiliations:** 1 Institute for Epidemiology, Kiel University, Kiel, Germany; 2 Scientific Institute of Diabetologists in Practice, Kaarst, Germany; 3 Department of Gynecology and Obstetrics, Vivantes Clinic Neukölln, Berlin, Germany; 4 Department of Obstetrics, Berlin Diabetes Center for Pregnant Women, St. Joseph Hospital, Berlin, Germany; 5 Department of Obstetrics, Competence Center for Diabetic Women, Jena University Hospital, Jena, Germany; 6 Saxony Center for Fetal-Neonatal Health, Faculty of Medicine and University Hospital Carl Gustav Carus, Technical University, Dresden, Germany; 7 Department of Obstetrics and Gynecology, University Hospital Schleswig-Holstein, Campus Kiel, Kiel, Germany; 8 Maternal Health and Midwifery Science, Julius Maximilians University of Würzburg, Würzburg, Germany; 9 Diabetes Center and Diabetes Education Center, Kiel, Germany

**Keywords:** COVID-19, Diabetes, Gestational, Pregnancy Outcome

## Abstract

**Introduction:**

Pregnancy is a known independent risk factor for a severe course of COVID-19. The relationship of SARS-CoV-2 infection and gestational diabetes mellitus (GDM) on neonatal outcomes is unclear. Our aim was to determine if SARS-CoV-2 infection represents an independent risk factor for adverse perinatal outcomes in pregnancy with GDM.

**Research design and methods:**

We compared data from two German registries including pregnant women with GDM, established during the SARS-CoV-2 pandemic (COVID-19-Related Obstetric and Neonatal Outcome Study (CRONOS), a multicenter prospective observational study) and already existing before the pandemic (German registry of pregnant women with GDM; GestDiab). In total, 409 participants with GDM and SARS-CoV-2 infection and 4598 participants with GDM, registered 2018–2019, were eligible for analyses. The primary fetal and neonatal outcomes were defined as: (1) combined: admission to neonatal intensive care unit, stillbirth, and/or neonatal death, and (2) preterm birth before 37+0 weeks of gestation. Large and small for gestational age, maternal insulin therapy, birth weight >4500 g and cesarean delivery were considered as secondary outcomes.

**Results:**

Women with SARS-CoV-2 infection were younger (32 vs 33 years) and had a higher median body mass index (28 vs 27 kg/m²). In CRONOS, more neonates developed the primary outcome (adjusted OR (aOR) 1.48, 95% CI 1.11 to 1.97) and were born preterm (aOR 1.50, 95% CI 1.07 to 2.10). Fasting glucose was higher in women in CRONOS versus GestDiab (5.4 vs 5.3 mmol/L) considering each 0.1 mmol/L increase was independently associated with a 5% higher risk of preterm birth among women in CRONOS only (aOR 1.05, 95% CI 1.01 to 1.09).

**Conclusions:**

GDM with SARS-CoV-2 infection in pregnancy is associated with an increased risk of adverse fetal and neonatal outcomes as compared with GDM without SARS-CoV-2 infection.

**WHAT IS ALREADY KNOWN ON THIS TOPIC**

- Current research suggests that a SARS-CoV-2 infection or COVID-19 increases the risk for a severe course of disease in pregnancy, affects maternal and neonatal outcomes, and is associated with an increase in gestational diabetes mellitus (GDM). However, information about the co-occurrence of SARS-CoV-2 infection and GDM in pregnant women is scarce.

**WHAT THIS STUDY ADDS**

- Neonates from women with gestational diabetes and SARS-CoV-2 infection are more likely to experience adverse perinatal outcomes; increasing fasting plasma glucose concentrations on the occasion of an oral glucose tolerance test (OGTT) appeared to be predictive for a worse neonatal outcome.

**HOW THIS STUDY MIGHT AFFECT RESEARCH, PRACTICE OR POLICY**

- Fetuses and newborns of women with gestational diabetes and SARS-CoV-2 infection or COVID-19 should receive enhanced surveillance because they present a vulnerable group, especially if vaccination coverage is low. Pregnant women and their offspring may benefit from vaccination against COVID-19.

## Introduction

Gestational diabetes mellitus (GDM) is one of the most common complications during pregnancy, and its prevalence has locally increased during the SARS-CoV-2 pandemic, associated with changes in lifestyle and modified GDM screening procedures.^1^ Lockdown measures and restrictions of social gatherings reduced the amount of physical activity and favored sedentary behavior, leading to adverse effects on pregnancy in women with GDM,^2^ such as excessive maternal gestational weight gain, worsening of glucose tolerance,^3^ and preterm birth.^4^ In addition, it is well known that increasing values from the oral glucose tolerance test (OGTT) are associated with increasing adverse perinatal outcomes.^5^

Uptake of SARS-CoV-2 occurs through its binding to the ACE2 receptor,^6^ which is more expressed by hyperinsulinemia.^7^ Even with subclinically elevated blood glucose concentrations, there is an increased structural glycation of the SARS-CoV-2 spike protein, facilitating its binding to the ACE2 receptor.^8 9^ From the upper respiratory tract, virus distribution is followed by systemic inflammation and entry into target organs, such as the endocrine pancreas.^10^ Moreover, specific placentitis leads to functional and morphological changes in the placenta with increased risk of stillbirth and neonatal death,^11^ predominantly associated with maternal viremia.^12^

Existing evidence suggests that COVID-19 increases the risk for adverse maternal and perinatal outcomes in pregnancy,^13–15^ and is associated with more GDM cases in pregnant women.^16^ We have previously reported that among unvaccinated pregnant women with COVID-19, GDM, particularly in combination with periconceptional overweight or obesity, was especially associated with adverse maternal outcomes.^17^ In the present analysis we would like to further evaluate the interrelation of GDM and SARS-CoV-2 infection focusing on fetal and neonatal outcomes.

To this end, we used data from two large national registries (see ‘Study samples’ section). Within the COVID-19-Related Obstetric and Neonatal Outcome Study (CRONOS), a multicenter maternity hospital-based registry study of SARS-CoV-2-infected pregnant women (covering the time period 2020–2022), we focused on women with GDM and SARS-CoV-2 infection. We compared the CRONOS data to data from the ‘GestDiab’ registry on women with GDM, a multicenter ongoing quality assessment study of specialized diabetologist offices, covering the time period between 2018 and 2019 before the pandemic.

Given the adverse impact of a SARS-CoV-2 infection on the course of diabetes, we hypothesized that, among women with GDM, the OGTT results would be increased in those infected with SARS-CoV-2 as compared with GDM cases before the pandemic, and that these differences in OGTT results will be associated with more adverse fetal and neonatal outcomes. Thus, the aims of our study were (1) to compare the odds of serious fetal and neonatal outcomes of SARS-CoV-2-infected versus non-infected pregnant women with GDM, (2) to determine differences in results of the OGTT between the two groups, and (3) to evaluate the association of OGTT results with the defined fetal and neonatal outcomes.

## Research design and methods

### Study samples

#### Sample of women with GDM and SARS-CoV-2 infection (subsample of the CRONOS cohort)

**Figure 1:**
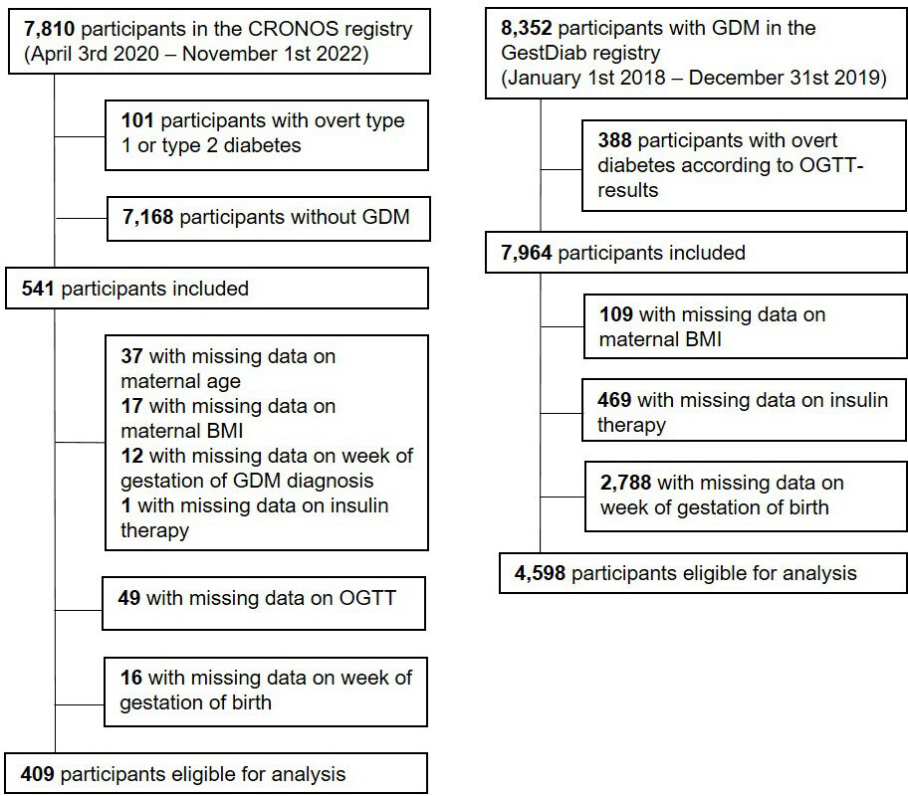
Flow chart showing the CRONOS and GestDiab participants being eligible for analysis.BMI, body mass index; CRONOS, COVID-19-Related Obstetric and Neonatal Outcome Study; GDM, gestational diabetes mellitus; OGTT, oral glucose tolerance test.

CRONOS is a multicenter prospective observational study cohort consisting of women with acute or previous SARS-CoV-2 infection during pregnancy, collected from 130 actively recruiting hospitals in Germany and Austria. The registry, whose methodology has been described elsewhere,^18^ was established by the German Society of Perinatal Medicine in April 2020. For the present analyses, we focused on women with GDM. A total of 7810 CRONOS participants underwent review and plausibility check, of which 409 women with GDM and SARS-CoV-2 infection from 35 centers were eligible for analysis (figure 1).

#### Sample of women with GDM before the pandemic and no SARS-CoV-2 infection (GestDiab registry)

The GestDiab registry collects information on fetal and neonatal outcomes in pregnant women with GDM. It is an ongoing quality assessment registry study by ‘winDiab’, the scientific institute of registered diabetologists. Diabetes specialist offices and diabetes outpatient clinics throughout Germany participate in GestDiab. The methodology of the GestDiab registry has been described previously.^19^ As a control group for the present analyses, we chose women with GDM between January 2018 and December 2019. In total, 4598 women with GDM from 81 centers were eligible for analysis (figure 1).

### Definition of GDM and GDM therapy

GDM was defined according to the ‘International Diabetes in Pregnancy Study Groups’ criteria.^20^ In Germany, a two-step approach is performed.^21^ First, a 50 g non-fasting 1-hour challenge test is performed between 24 and 28 weeks of pregnancy. Women with a test result >7.5 mmol/L require a 75 g OGTT. GDM is confirmed if any of the following venous plasma glucose values are met or exceeded: fasting: 5.1 mmol/L, 1 hour: 10.0 mmol/L, and 2 hours: 8.5 mmol/L. Cases and controls with documentation of all three glucose values of the OGTT were eligible for the present analysis.

According to the German guidelines, GDM treatment with insulin is indicated when more than 50% of self-monitored capillary blood glucose results within 1–2 weeks exceed 5.3 mmol/L fasting and 7.8 mmol/L 1 hour or 6.7 mmol/L 2 hours after a main meal. To guide the intensity of treatment and to detect fetal macrosomia, fetal growth is regularly monitored by ultrasound examinations.^21^

### Treatment protocol of COVID-19

The care and treatment of the pregnant women was carried out from the local caregivers according to the joint German, Austrian, and Swiss COVID-19 guidelines for pregnant women.^22^

### Outcome definition

All fetal and neonatal outcomes of interest were specified a priori to avoid outcome reporting bias. We prespecified two primary outcomes: (1) combined: admission to the neonatal intensive care unit (NICU) or stillbirth and/or neonatal death, (2) preterm birth ≤37+0 weeks of gestation. Neonatal death was defined as death of a liveborn newborn who deceased within 7 days after birth. Secondary outcomes were prespecified as (3) large for gestational age (LGA), classified as birth weight >90th centile for gestational age and sex, (4) small for gestational age (SGA), classified as birth weight <10th centile for gestational age and sex, (5) maternal insulin therapy, (6) cesarean delivery, and (7) birth weight ≥4500 g. Of note, the components of both the primary and secondary outcomes were collected using identical criteria in both registries. For the main analysis, women with missing data on the combined primary outcome (CRONOS n=8, GestDiab n=669) were excluded. Missing data for the outcome preterm birth were already excluded as criteria in the selection of the study population (CRONOS n=16, GestDiab n=2788; figure 1).

### Definition of virus variants

SARS-CoV-2 infection in this article refers to laboratory test-confirmed symptomatic cases (defined as COVID-19) and laboratory test-confirmed SARS-CoV-2 infection without symptoms. Based on different criteria,^23 24^ the Robert Koch Institute defined the dominant virus strain in each phase of the pandemic. Based on the infection date, the pregnant women within the CRONOS registry were assigned to these periods and to the dominant virus strain.^23^

### Statistical methodology

Statistical analyses were conducted using SAS software (V.9.4, SAS Institute). Sample size estimation was performed using R (V.4.3.2) and RStudio (build 446). P values <0.05 were considered statistically significant. Holm-Bonferroni correction was applied to account for multiple testing in the two primary outcomes. To estimate potential selection bias, characteristics of the final analytical sample were compared with those of excluded participants (online supplemental tables 6 and 7). Results are presented as means±SD for normally distributed data and median (Q_25_; Q_75_) for non-normally distributed continuous data. Means and medians of the two samples were compared using Student’s t-test (equal variances) or Welch’s t-test (unequal variances) for normally distributed continuous variables or Mann-Whitney U test for continuous non-normally distributed variables, respectively. To compare categorical variables the χ^2^ test and Fisher’s exact test were used.

10.1136/bmjdrc-2023-003724.supp1 Supplementary data

### Association of SARS-CoV-2 infection status with fetal and neonatal outcomes and venous plasma glucose concentrations

Multivariable-adjusted logistic regression models were used to analyze the associations of SARS-CoV-2 infection status (yes vs no; independent variable) with the primary and secondary outcome variables (dependent variable), and linear regression models (analysis of covariance) were used to compare the venous plasma glucose concentrations at different time points (fasting, 1 hour, 2 hours; dependent variable) from the OGTT between women with GDM with versus without SARS-CoV-2 infection (independent variable).

### Associations of venous plasma glucose concentrations from OGTT with fetal and neonatal outcomes

In each sample (women with vs without SARS-CoV-2), the associations of venous plasma glucose concentrations from OGTT with continuous traits and with binary outcome variables (primary and secondary outcomes) were examined using multivariable-adjusted logistic regression analysis. Interactions in the association of blood glucose concentrations with the outcomes between women with (CRONOS) and without (GestDiab) SARS-CoV-2 infection were examined by adding a corresponding interaction term to the logistic regression models. A logistic regression model was calculated, stratified by registry in case of a statistically significant interaction.

To control for potential confounding, all of the above described models were adjusted for maternal body mass index (BMI), maternal age, gestation week of GDM diagnosis, and maternal insulin therapy (yes/no). Fasting venous blood glucose concentration was added as a confounder in selected relevant models.

In the cohort of women with SARS-CoV-2 infection (CRONOS), some additional analyses were conducted: we assessed the associations between venous plasma glucose concentrations from OGTT (continuous dependent variable in separate models) and perinatal primary outcomes with additional adjustment for the sequence of infection (diagnosis of SARS-CoV-2 infection or GDM first), the SARS-CoV-2 virus variant type of concern (pre-Omicron vs Omicron), and vaccination status (yes/no). In addition, we analyzed the frequency of primary outcomes depending on the severity of the maternal infection. A severe maternal course of COVID-19 was defined as a combination of intensive care unit (ICU) admission, viral pneumonia and oxygen supplementation.

### Sample size calculation

We performed a sample size calculation on preliminary data of the CRONOS cohort regarding the analysis. The estimated needed overall sample size is at least 1051 observations to be sufficient to detect a difference of at least 10% with alpha of 0.05 and power of 90% with respect to the primary outcomes.

## Results

### Comparison of women with GDM with versus without SARS-CoV-2 infection

Women with GDM and SARS-CoV-2 infection (CRONOS sample) were younger (32 vs 33 years) and had a higher median BMI (28 vs 27 kg/m²) as compared with women with GDM and without SARS-CoV-2 infection (GestDiab sample) (table 1). Among CRONOS in almost three-quarters (71.2%) of participants, the diagnosis of SARS-CoV-2 infection was confirmed concurrently with or shortly after the GDM diagnosis, in 28.8% before GDM diagnosis. The majority of women (80.9%) showed COVID-19-related symptoms, and about one-fifth (21.4%) of the participants were vaccinated against COVID-19 at least once since vaccination was available (table 2).

**Table 1:** Characteristics of the study samples of women with GDM with (CRONOS) and without (GestDiab) SARS-CoV-2 infection

Characteristic	n	Cohort with SARS-CoV-2 n=409	Cohort without SARS-CoV-2 n=4598	P value
**Maternal basic data and outcomes**				
Maternal age (years)	409/4598	32 (28; 36)	33 (29; 36)	**0.001**
Maternal BMI (kg/m^2^)		28.0 (24.2; 33.1)	27.0 (23.3; 32.0)	**0.006**
Week of gestation of gestational diabetes mellitus diagnosis		26 (24; 28)	26 (25; 28)	0.152
Insulin therapy, n (%)		148 (36.2)	1474 (32.1)	0.087
BMI categories according to WHO, n (%)				
Underweight (<18.5 kg/m²)	409/4598	8 (1.96)	65 (1.4)	**0.007**
Normal weight (18.5–24.9 kg/m²)		111 (27.1)	1659 (36.1)	
Preobesity (25.0–29.9 kg/m²)		131 (32.0)	1322 (28.8)	
Obesity class I (30.0–34.9 kg/m²)		89 (21.8)	852 (18.5)	
Obesity class II (35.0–39.9 kg/m²)		37 (9.1)	430 (9.4)	
Obesity class III (≥40 kg/m²)		33 (8.1)	270 (5.9)	
Obesity class I, II, III		159 (38.9)	1553 (33.8)	**0.037**
Diagnostic venous plasma glucose during OGTT				
Fasting (mmol/L)†		5.4±0.5	5.3±0.5	**0.0001**
After 1 hour (mmol/L)†		9.5±1.7	9.6±1.8	0.664
After 2 hours (mmol/L)†		7.5±1.5	7.5±1.5	0.778
Only at fasting, n (%)		177 (43.3)	1844 (40.1)	0.210
At all 3 time points, n (%)		45 (11)	466 (10.1)	0.579
Cesarean delivery, n (%)	408/4390	173 (42.4)	1554 (35.4)	**0.005**
**Neonatal outcomes**				
Combined (NICU admission, neonatal death, stillbirth), n (%)	401/3929	63 (15.7)	431 (11.0)	**0.004**
Preterm birth <34+0 weeks of pregnancy, n (%)	409/4598	15 (3.7)	78 (1.7)	**0.005**
Preterm birth <37+0 weeks of pregnancy, n (%)	409/4598	43 (10.5)	342 (7.4)	**0.025**
Large for gestational age, n (%)	368/4395	48 (13.0)	611 (13.9)	0.647
Small for gestational age, n (%)		28 (7.6)	343 (7.8)	0.893
Birth weight <2500 g, n (%)	404/4509	31 (7.7)	200 (4.4)	**0.003**
Birth weight >4000 g, n (%)		53 (13.1)	543 (12.0)	0.526
Birth weight >4500 g, n (%)	404/4509	8 (2.0)	70 (1.6)	0.510
Apgar 5 <7, n (%)	403/2813	18 (4.5)	37 (1.3)	**0.0001**
Umbilical arterial cord pH <7.1, n (%)	398/2573	22 (5.5)	89 (3.5)	**0.043**
NICU admission, n (%)	399/4177	58 (14.5)	423 (10.1)	**0.006**
Stillbirth, n (%)	406/4525	5 (1.2)	8 (0.2)	**0.003**
Neonatal death, n (%)	401/4189	2 (0.5)	0 (0)	**0.008**

Data are presented as number/total number (percentage) or mean±SD, unless otherwise specified.

P values <0.05 are for comparison between the CRONOS and GestDiab registry based on the χ^2^ test or Fisher’s exact test (categorical variables) or Student’s t-test (continuous normally distributed variables) or Mann-Whitney U test for normally distributed continuous variables or for continuous non-normally distributed variables, respectively.

The bold p-values indicate statistical significance.

*Median and IQR.

†To convert to mg/dL multiply mmol/L with 18.02.

BMI, body mass index; CRONOS, COVID-19-Related Obstetric and Neonatal Outcome Study; GDM, gestational diabetes mellitus; NICU, neonatal intensive care unit; OGTT, oral glucose tolerance test.

**Table 2:** CRONOS study cohort

Maternal characteristic	n	Cohort with SARS-CoV-2 n=409
COVID-19 with/after GDM diagnosis	409	291 (71.2%)
COVID-19 assessment tool	409	PCR test 365 (89.2%); test positive, tool unknown 26 (6.4%); antigen test 14 (3.4%); antibody test 4 (1.0%)
COVID-19-associated symptoms	402	325 (80.9%)
Vaccinated after availability since January 2021	383	82 (21.4%)
Additional care from diabetologists	386	327 (84.7%)
Virus pneumonia	404	20 (5.0%)
Intensive care unit admission	405	15 (3.7%)
Intubation	404	6 (1.5%)
Mother deceased	405	0 (0%)
Oxygen supplementation	405	24 (5.9%)
COVID-19 treatment	366	Glucocorticoid 5 (1.4%), antiviral agents 1 (0.3%), monoclonal antibodies 1 (0.3%), others 4 (1.1%)
Virus variants of concern (VOC)*	409	VOC Omicron 141 (34.5%), Wild type 127 (31.1%), VOC Delta 81 (19.8%), VOC Alpha 51 (12.5%), VOC Omicron BA5 2 (0.5%)
5 most commonly reported COVID-19-related symptoms in symptomatic cases	344	Cough 173 (50.3%), malaise 140 (40.7%), fatigue 132 (38.4%), sore throat 126 (36.6%), headache 120 (34.9%)

Data are presented as number/total number (percentage).

*Classification based on the definition of the dominant virus strain in each phase of the pandemic from Robert Koch Institute.^23^

CRONOS, COVID-19-Related Obstetric and Neonatal Outcome Study; GDM, gestational diabetes mellitus.

### Associations of SARS-CoV-2 infection with adverse perinatal outcomes

**Figure 2:**
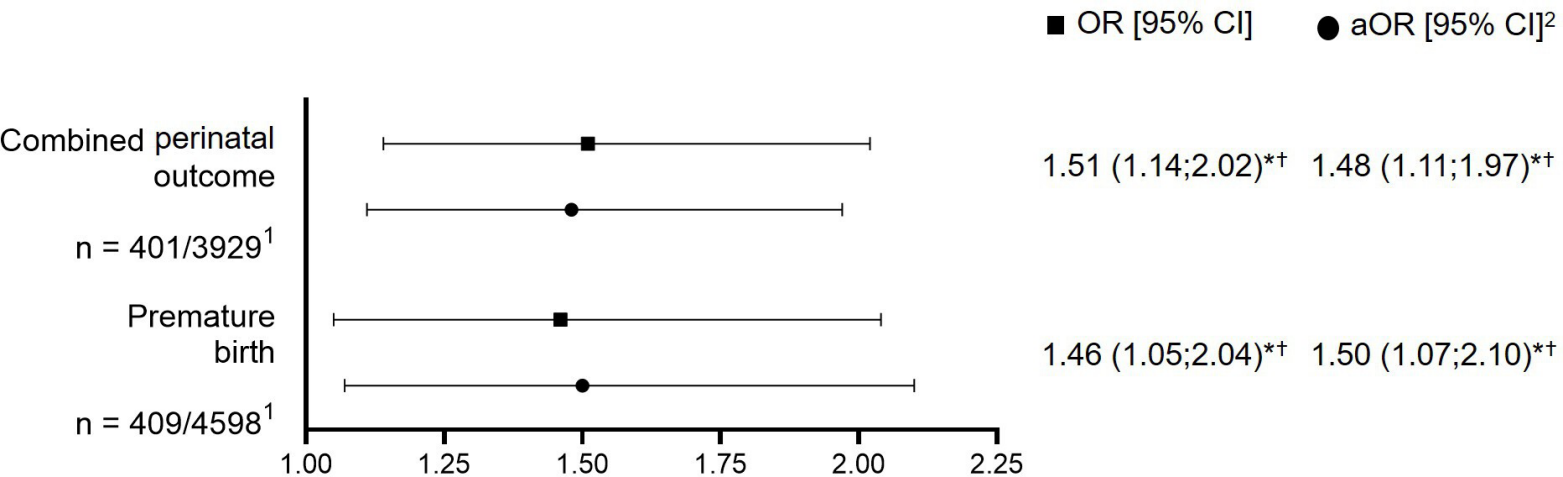
Comparison of the odds for primary adverse neonatal outcomes between women with gestational diabetes mellitus (GDM) with (COVID-19-Related Obstetric and Neonatal Outcome Study, CRONOS) and without (GestDiab) SARS-CoV-2 infection. Data are presented as adjusted OR (aOR) (95% CI) using logistic regression analyses for the two primary neonatal outcomes: combined neonatal outcome (admission to neonatal intensive care unit, stillbirth, and/or neonatal death) and preterm birth (yes or no) as the dependent variable (separate model for each). *P<0.05. †Holm-Bonferroni corrected for multiple testing. ^1^CRONOS/GestDiab. ^2^Adjusted for maternal body mass index (BMI), maternal age, gestation week of GDM diagnosis, insulin therapy, and fasting blood glucose concentration. OGTT, oral glucose tolerance test.

The adjusted OR (aOR) to develop the combined primary outcome was statistically higher among women with GDM and SARS-CoV-2 infection as compared with women with GDM before the pandemic (aOR 1.48, 95% CI 1.11; 1.97), as was the OR for preterm birth (aOR 1.50, 95% CI 1.07; 2.10; figure 2). Women with GDM, SARS-CoV-2 infection and a severe course of COVID-19 (ie, maternal transfer to ICU, invasive ventilation, oxygen supply) had higher odds to develop the combined primary outcome (aOR 7.11, 95% CI 3.04; 16.60) or preterm birth (aOR 4.42, 95% CI 1.64; 11.90) compared with those women with no severe course of disease (online supplemental table 1). Analysis of the different virus variants showed that there was no difference between the variants of concern in the odds of developing the combined outcome (aOR 1.63, 95% CI 0.87; 3.03) and preterm birth (aOR 0.89, 95% CI 0.44; 1.81) between women infected by SARS-CoV-2 during the time with predominance of the Omicron variant versus pre-Omicron variants. However, during the pre-Omicron period, in neonates the risk to be born LGA was increased compared with the Omicron period (aOR 2.79, 95% CI 1.33; 5.84, online supplemental table 2).

Regarding the secondary outcomes, the adjusted odds for cesarean delivery (aOR 1.33, 95% CI 1.08; 1.64) were higher in women with GDM and SARS-CoV-2 infection as compared with women with GDM before the pandemic, while no differences were observed for LGA, SGA, maternal insulin therapy, and birth weight ≥4500 g (online supplemental table 3).

### Blood glucose concentrations and their associations with adverse neonatal outcomes

**Figure 3:**
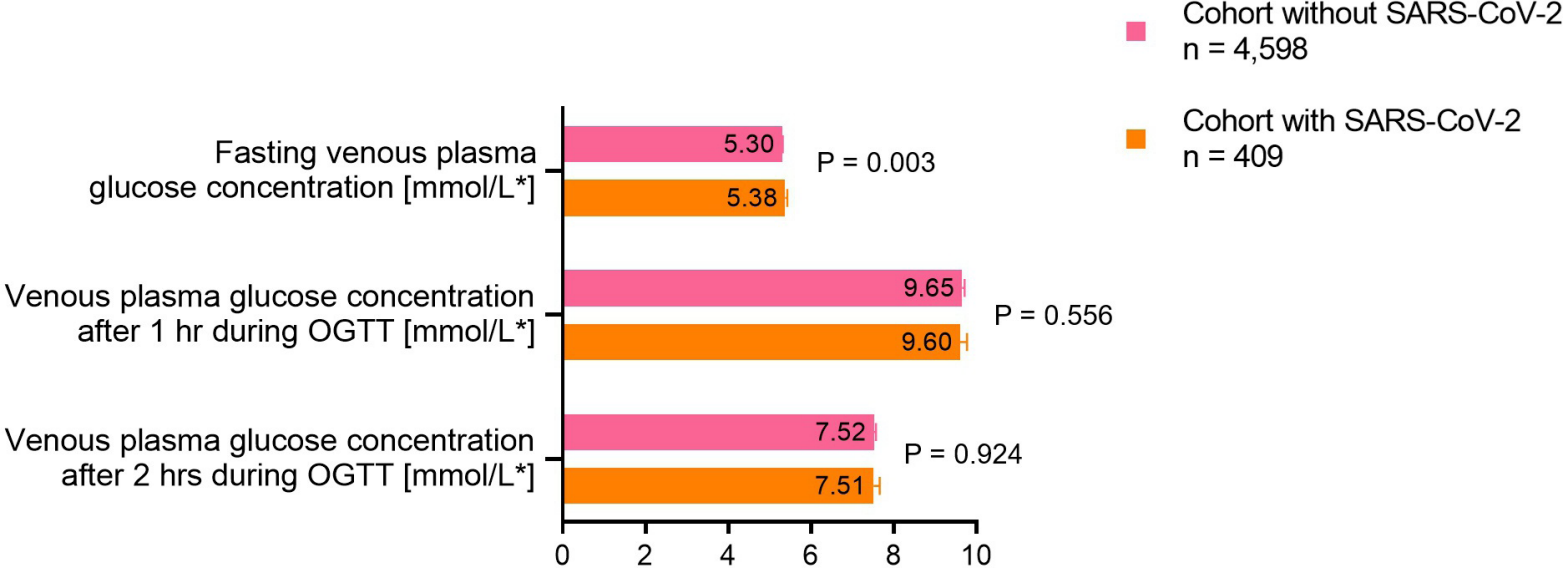
Comparison of adjusted oral glucose tolerance test (OGTT) results between women with gestational diabetes mellitus (GDM) with (COVID-19-Related Obstetric and Neonatal Outcome Study, CRONOS) and without (GestDiab) SARS-CoV-2 infection. Data are presented as mean [SEM], adjusted for maternal body mass index (BMI), maternal age, week of gestation of GDM diagnosis, and insulin therapy. *To convert to mg/dL multiply mmol/L with 18.02. OGTT, oral glucose tolerance test.

Fasting, but not postprandial venous plasma glucose, was statistically significantly higher among women with GDM and SARS-CoV-2 infection as compared with women with GDM before the pandemic (5.4 vs 5.3 mmol/L; p=0.003; figure 3). Each 0.1 mmol/L increment in fasting venous plasma glucose was associated with 5% higher OR for preterm birth (aOR 1.05, 95% CI 1.01; 1.09), but only among women with GDM and SARS-CoV-2 infection (CRONOS cohort; online supplemental table 4), while there was no statistically significant difference between the two registries in neither the association of postprandial glucose concentrations with preterm birth nor in the association of the combined outcome with any of the blood glucose concentrations from OGTT (data not shown).

### Associations of blood glucose with adverse perinatal outcomes among women with GDM and SARS-CoV-2 infection

When adjusted for COVID-19-related confounders (COVID diagnosis after GDM diagnosis, variant type, vaccination status), a higher fasting blood glucose concentration was associated with higher odds for the combined primary outcome (aOR 1.04, 95% CI 1.00; 1.07) and for preterm birth (aOR 1.05, 95% CI 1.01; 1.09), among women with GDM and SARS-CoV-2 infection (CRONOS cohort; online supplemental table 5).

### Comparison of excluded participants with the final analytical sample

When comparing the final analytical sample of women with GDM and SARS-CoV-2 infection to those women who were excluded due to incomplete data, the excluded participants had a higher proportion of maternal insulin therapy (54.8% vs 36.2%, p=0.002) and had an earlier diagnosis of GDM (25 vs 26 weeks of pregnancy, p=0.005) (online supplemental table 6).

Among women with GDM before the pandemic, no difference was observed for the median BMI, week of gestation of GDM diagnosis, and prevalence of obesity between the analytical sample and those women who had to be excluded due to missing data. However, excluded participants were slightly younger (32 vs 33 years, p=0.02) and had a lower rate of maternal insulin therapy (24.6% vs 32.1%, p=0.0001) compared with the analytical sample (online supplemental table 7).

## Discussion

In this study, our main observation was that neonates from women with GDM and SARS-CoV-2 infection were more likely to experience adverse perinatal outcomes (ie, NICU admission, neonatal death, and/or stillbirth) and to be born preterm compared with neonates born from women with GDM before the SARS-CoV-2 pandemic. In addition, SARS-CoV-2 infection in women with GDM resulted in a higher proportion of cesarean deliveries as compared with women with GDM before the pandemic. When comparing OGTT results, women’s fasting levels were higher in cases with SARS-CoV-2 infection and solely increased fasting plasma glucose—but not postchallenge levels—was associated with a higher risk of preterm birth. This association was only observed in women with GDM and SARS-CoV-2 infection, but not in women with GDM before the pandemic.

### SARS-CoV-2 infection in GDM and risk of adverse neonatal outcomes

Some prior studies have reported an increased risk of preterm birth in neonates of mothers with COVID-19 during pregnancy,^15 25–29^ and we can confirm this association in women with GDM. There are several possible obstetrical reasons for preterm birth. Severe COVID-19 late in pregnancy could worsen the mother’s health condition, followed by multiorgan disease from viremia including placentitis in severe cases, and worsening of oxygen saturation. This, in turn, could lead to acute fetal distress, increasing the rate of emergency cesarean delivery and preterm birth due to fetal indication or more liberally general indication in the early period of the pandemic. Supporting the hypothesized underlying mechanism, here, increase in risk of preterm birth and the combined perinatal outcome particularly appeared in severe course of COVID-19, probably associated with metabolic imbalances and increasing blood glucose from GDM. Additionally, we observed an increased rate of LGA in the early waves of the pandemic compared with the Omicron period. More sedentary behavior, changed eating habits, and excessive gestational weight gain could contribute to higher blood glucose in the pregnant women, followed by increased transplacentary mother-to-fetus glucose transport, consecutive fetal hyperinsulinemia and insulin-mediated stimulation of fetal growth. Nevertheless, based on billing data of pregnant women covered by German statutory health insurance, it was possible to determine that prenatal care and GDM screening was also used intensively in the first year of the pandemic.^30^ With no data from the years 2021–2022, however, it cannot be ruled out that personal appointments may have been less frequent, possibly contributing to overlooked fetal growth acceleration.

### OGTT results and risk for adverse neonatal outcomes

Levels of fasting plasma glucose were higher in women with GDM and SARS-CoV-2 infection compared with those with GDM before the pandemic; the majority of the infected women were symptomatic with COVID-19-related symptoms. Similar results were recently published by others.^31^ In our study, for most women the SARS-CoV-2 infection was diagnosed with or shortly after GDM was confirmed. Fasting hyperglycemia per se is associated with pronounced insulin resistance and consecutive hyperinsulinemia,^32^ which could facilitate virus entry and distribution. On the other hand, COVID-19 could contribute to increased blood glucose levels through systemic inflammation and oxidative stress. In the comparison of women with GDM with and without SARS-CoV-2 infection, fasting glucose results from OGTT were associated with adverse perinatal outcomes despite GDM therapy in accordance with the German guidelines. Associations of increasing glucose levels from OGTT with adverse perinatal outcomes are well known from pregnant women with GDM without treatment.^5^ In general, the risks of perinatal complications in treated women with GDM are associated with trajectories of glycemic control, depending on, for example, (1) how fast glucose control can be improved, (2) how long optimal control is maintained between GDM diagnosis and birth,^33^ and (3) the used glycemic targets.^34^

### Vaccination status

In the CRONOS cohort 21.4% of women with GDM received at least one vaccination dose against COVID-19 since its availability in January 2021, which is far below the German population basic immunization rate of 85.4% up to November 2022,^35^ the time point of CRONOS data extraction. Vaccination against COVID-19 during pregnancy is safe and highly effective, not associated with higher than average rate of side effects, and reduces the risk of stillbirth, preterm birth, and NICU admission.^36 37^ Future research should evaluate the effect of vaccination against COVID-19 on maternal and neonatal outcomes in women with GDM. Many pregnant women are still reluctant to be vaccinated against COVID-19,^38^ so they should be counseled with support of more specific information on vaccination and be motivated to take part in the recommended vaccination program receiving benefits for themselves and their offspring. Under the recent Omicron variants maternal and neonatal risks are still of concern in symptomatic and unvaccinated women.^39^ Furthermore, there are currently no reliable findings on post-COVID-19 condition after GDM,^40^ whether COVID-19 during pregnancy accelerates the future risk of type 2 diabetes in the mother, and whether COVID-19 is associated with any long-term increase of risks in the exposed offspring.

### Strengths and limitations

The strengths of our study are as follows: We used data from high-quality managed homogenous cohorts with frequent data monitoring. Additionally, in CRONOS, validation recalls with each local center concerning confirmation of SARS-CoV-2 infection, GDM diagnosis, insulin therapy, and pregnancy outcomes were carried out to detect and eliminate discrepancies. GDM was confirmed with OGTT results from both registries to avoid inaccuracy from International Classification of Diseases coding, hence cases with overt diabetes and misdiagnosis (no GDM) could certainly be excluded.

Some limitations merit consideration. First, data were collected in different time frames, each at least of 2 years’ duration. During these time periods, screening and management of GDM, treatment guidelines of SARS-CoV-2 infection or vaccination rates against COVID-19 may have changed, and the proportion of obesity, levels of stress and anxiety might have increased. In addition, before the pandemic, it had been extremely uncommon that women with GDM were transferred to ICU, received invasive ventilation or oxygen supply, so that these items were not included in the GestDiab dataset and could therefore not be included as covariates in our analyses. Second, the registry data were recruited in outpatient and hospital settings and therefore comparison has some residual restrictions. Third, in GestDiab, pregnancy outcome data were obtained in the diabetes outpatient offices either at the first postpartum visit or from discharge letters from maternity hospitals. Since only 38.2% of mothers attended the first postpartum visit,^19^ this might have accounted for the proportion of excluded participants. However, comparing the analyzed cohorts with the excluded women due to missing data, excluded women in CRONOS were earlier diagnosed with GDM and were more frequently managed with insulin. In contrast, excluded cases in GestDiab were younger and received less often insulin. From this observation, we can assume that the effect size of GDM combined with SARS-CoV-2 infection on the fetal and neonatal outcomes in our analysis may be underestimated. Fourth, because of different coding, chronic hypertension or pre-eclampsia could not be reliably differentiated in both registries and therefore were not included in the analysis. Lastly, data on the quality of diabetes management after GDM diagnosis were not available; targeting glucose control and duration of optimal control may be associated with improved outcomes.

In conclusion, neonates from women with GDM and SARS-CoV-2 infection were more likely to experience adverse perinatal outcomes, especially NICU transfer, stillbirth, and neonatal death, and were more frequently born preterm compared with neonates born to women with GDM before the SARS-CoV-2 pandemic. In addition, the higher fasting plasma glucose concentrations among women with SARS-CoV-2 infection appeared to be predictive for a worse perinatal outcome. Thus, with regard to the new phase of SARS-CoV-2 variants spread, fetuses and newborns of women with GDM and SARS-CoV-2 infection should still receive attention as a vulnerable group particularly if vaccination coverage is low.

## Data Availability

No data are available. Not provided due to data protection reasons.
