# Supplementary material for: Adverse perinatal outcomes in gestational diabetes mellitus with and without SARS-CoV-2 infection during pregnancy: results from two nationwide registries in Germany
Source: BMJ Open Diabetes Res Care. 2024 Jan 25;12(1):e003724. doi: 10.1136/bmjdrc-2023-003724 (PMC10823927; doi:10.1136/bmjdrc-2023-003724)
Supplement: Supplementary data [file bmjdrc-2023-003724supp001.pdf]

SUPPLEMENTARY MATERIAL

Study Collaborators

Local collaborators in CRONOS

Babett Ramsauer, MD (Berlin), Tanja Groten, MD (Jena), Ute Schaefer-Graf, MD (Berlin), Titzian Jahreis, MD (Memmingen), Nora Horn, MD (Berlin), Laura Lüber, MD (Ravensburg), Luise Gattung MD (Bad Salzungen), Eva Pecqueux, MD (Dresden), Angela Lihs, MD (Stuttgart), Iris Dressler-Steinbach, MD (Berlin), Michael Bohlmann, MD PhD (Lörrach), Clara Backes, MD (München), Monika Palz-Fleige, MD (Dortmund), Julia Günther, MD (Hannover), Sven Kehl, MD (Erlangen), Jula Manz, MD (Darmstadt), Lydia Remtisch, MD (Leipzig), Maria Delius, MD (München), Dörthe Brüggmann, MD PhD (Frankfurt), Annemarie Minte, MD (Coesfeld), Valerie Meister, MD (Starnberg), Kathleen Sondern, MD (Münster), Filiz Markfeld-Erol, MD (Freiburg), Christine Morfeld, MD (Hannover), Elsa Hollatz-Galuschki, MD (Nürnberg), Anja Leonhardt (Chemnitz), Anja Jebens, MD (Berlin), Mark Dzietko, MD (Essen), Nina Axnick (Berlin), Constanze Banz-Jansen, MD PhD (Bielefeld), Anna Treptow, MD (Dresden), Olaf Parchmann, MD (Sangerhausen), Agna Ozalinskaite, MD (Bayreuth), Sven Seeger, MD (Halle), Johannes Stuber, MD PhD (Rostock), Thomas Müller, MD PhD (Hanau), Siegmund Köhler, MD (Marburg), Markus Schmidt, MD PhD (Duisburg), Nadine Mand, MD (Marburg), Marcel Malan, MD (Hamburg), Ulrich Pecks, MD (Kiel)

Local collaborators in GestDiab

Heinke Adamczewski, MD (Köln), Kirsten Holtappels, MD (Neuss), Bidjan Massoudy, MD (Bonn), Manfred Kurnoth, MD (Mönchengladbach), Susanne Schmidt, MD (Alsdorf), Ralph Bierwirt, MD (Essen), Burkhard Schmidt, MD (Wegberg), Jamal Sobh, MD (Siegburg), Ellen Zavaleta, MD (Köln), Eugen Steffens, MD (Köln), Cosima Schramm, MD (Jena), Nikolaus Scheper, MD (Marl), Eva Hess, MD (Worms), Matthias Kaltheuner, MD (Leverkusen), Martina Pothe-Sarabhai, MD(Düsseldorf),Peter Puth, MD (Kamen), Michael Simonsohn, MD (Frankfurt/Main), Petra Ferber, MD (Euskirchen), Hubertus Halbfas, MD (Bergisch Gladbach), Anita Kortemeier, MD (Iserlohn), Stephan Schleyer, MD (Wetter), Rudolf Groddeck, MD (Dortmund), Mathias Bohle, MD (Hamm), Silke Fröhlich (Senden), Hansjörg Mühlen, MD (Duisburg), Maria Gumprich, MD (Wuppertal), Herbert Lutz, MD (Ludwigshafen), Ralf Kolassa, MD (Bergheim), Gabriele Wildemann-Gilbert, MD (Karlsruhe), Kirsten Hellner, MD (Hamburg), Christian Malcharzik, MD (Hannover), Matthias Riedel, MD (Köln), Judith Nagel, MD (Köln), Ingrid Rein, MD (Warendorf), Michael Esser, MD (Essen), Marie-Therese Schäfermeyer, MD (Greven), Carsten Volkery, MD (Minden), Fabian Scholz, MD (Berlin), Michael Lang, MD (Dortmund), Babette Lorra, MD (Bochum), Ariane Sellmann, MD (Mülheim), Jörn Kugler, MD (Köln), Dorothea Reichert, MD (Landau), Payam Ardiomand, MD (Bergisch Gladbach), Stephan Kern, MD (Bonn), Gernot Sachs, MD (Hückelhoven), Ernst-Otto von Reis, MD (Duisburg), Udo Kallenberg, MD (Stolberg), Michael Naudorf, MD (Lindlar), Tobias Ohde, MD (Essen), Michael Kann, MD (Ruppach-Goldhausen), Winfried Keuthage, MD (Münster), Guido Schomacher, MD (Rheine), Helga Zeller-Stefan, MD (Essen), Cornelia Woitek, MD (Wurzen), Carsten Ianello, MD (Mosbach), Jolanda Schottenfeld-Naor, MD (Düsseldorf), Dorothea Gronwald, MD (Duisburg), Ingo Knipp, MD (Himmelpforten), Rosemarie Weber-Lauffer, MD (Karlsruhe), Ralf Barion, MD (Niederkassel), Simone Badis, MD (Wittlich), Ingrid Mahle, MD (Freudenstadt), Jasmin Ruth, MD (Velbert), Katrin Liebich, MD (Berlin), Antje Weichard, MD (Magdeburg), Jost Hilgenberg, MD (Rehburg-Loccum), Martin Schuster, MD (Solingen), Urs Schaden (Düsseldorf), Barbara Sawitzky-Rose, MD (Berlin), Christoph Heyer, MD (Viersen), Martin Puschmann, MD (Gummersbach), Robert Ostermann-Myrau, MD (Dormagen), Matthias Schilling, MD (Manderscheid), Young Hee Lee-Barkey, MD (Bad Oeynhausen), Hannelore Klimke, MD (Mettmann), Jochen Schmidt-Walczuch (Brühl), Carsten Klugewitz, MD (Essen), Ulrike Brockmann, MD (Bedburg), Andreas Haman, MD (Bad Nauheim)

Supplementary Table 1- Comparison of the combined primary outcome and premature birth in women with a severe course of disease vs. women without a severe course of disease within the sample of women with GDM and SARS-CoV-2 infection (CRONOS-cohort)

	n (column %)	Severe course of COVID-19 (n [row %])	No severe course of infection (n [row %])	Crude	Multivariable
Variable				OR [95% CI]	aOR [95% CI]*
Combined primary outcome					
No severe SARS-CoV-2 infection/ COVID-19	370 (93.4%)	50 (13.5%)	320 (86.5%)	Ref	Ref
Severe COVID-19	26 (6.6%)	13 (50%)	13 (50%)	**6.40 [2.81;14.60]**	**7.11 [3.04;16.60]**
Premature birth					
No severe SARS-CoV-2 infection/ COVID-19	378 (93.6%)	36 (9.5%)	342 (90.5%)	Ref	Ref
Severe COVID-19	26 (6.4%)	7 (26.9%)	19 (73.1%)	**3.50 [1.38;8.89]**	**4.42 [1.64;11.90]**

Data are presented as number/total number (percentage) and OR/aOR (95% CI) using logistic regression analyses.

*adjusted for maternal BMI, maternal age, gestation week of GDM diagnosis, insulin therapy, fasting blood glucose concentration, and week of gestation at the onset of COVID-19 symptoms

Supplementary Table 2- Comparison of primary neonatal outcomes, LGA, and insulin therapy in CRONOS between pre-omicron period (wildtype/alpha/delta) vs. omicron period (omicron/omicron BA5)

	n	Pre-omicron period	Omicron period	Crude	Multivariable
Variable				OR [95% CI]	aOR [95% CI]*
Combined primary neonatal outcome	253/141	45 (17.8%)	17 (12.1%)	1.58 [0.87;2.88]	1.63 [0.87;3.03]
Premature birth	259143	27 (10.4%)	15 (10.5%)	0.99 [0.51;1.94]	0.89 [0.44;1.81]
LGA	223/138	37 (16.6%)	11 (8.0%)	**2.30 [1.13;4.67]**	**2.79 [1.33;5.84]**
Insulin therapy	259/143	91 (35.1%)	55 (38.5%)	0.87 [0.57;1.32]	0.88 [0.56;1.38]

Data are presented as number/total number (percentage) and OR/aOR (95%CI) using logistic regression analyses.

*adjusted for maternal BMI, maternal age, gestation week of GDM diagnosis, insulin therapy, fasting blood glucose concentration, and week of gestation at the onset of COVID-19 symptoms

LGA, large for gestational age

Supplementary Table 3- Comparison of secondary outcomes between women with GDM with (CRONOS) and without (GestDiab) SARS-CoV-2 infection

	Crude	Multivariable
Variable	OR [95% CI]	aOR [95% CI]*
LGA	1.08 [0.79;1.48]	1.15 [0.83;1.58]
SGA	0.97 [0.65;1.45]	1.02 [0.68;1.53]
Caesarean delivery	**1.34 [1.09;1.65]**	**1.33 [1.08;1.64]**
Insulin therapy (maternal)	1.20 [0.97;1.48]	1.19 [0.96;1.48]
Birth weight $\geq$ 4500g	1.28 [0.61;2.68]	1.17 [0.56;2.45]

Data are presented as OR/aOR (95% CI) using logistic regression analyses for the five secondary outcomes: LGA, SGA, caesarean delivery, insulin therapy, birth weight $\geq$4500g (yes or no) as the dependent variable (separate model for each).

*adjusted for maternal BMI, maternal age, gestation week of GDM diagnosis, insulin therapy, and fasting blood glucose concentration

SGA, small for gestational age, LGA, large for gestational age

Supplementary Table 4- Associations (interactions) between OGTT results and preterm birth between women with GDM with (CRONOS) and without (GestDiab) SARS-CoV-2 infection

		Cohort with SARS-CoV-2		Cohort without SARS-CoV-2		
	Variable	aOR [95% CI]*	P	aOR [95% CI]*	P	P of interaction
Preterm birth	Fasting venous plasma glucose concentration [mmol/L]*	1.05 [1.01;1.09]	**0.019**	1.00 [0.99;1.01]	0.383	**0.007**
	Venous plasma glucose concentration after 1 hr [mmol/L]**	1.01 [1.00;1.02]	0.079	1.00 [1.00;1.01]	0.076	0.358
	Venous plasma glucose concentration after 2 hrs [mmol/L]**	1.01 [1.00;1.02]	0.238 0.199	1.01 [1.00;1.01]	**0.008**	0.984

Data are presented as aOR (95% CI) using logistic regression.

*adjusted for maternal BMI, maternal age, week of gestation of gestational diabetes mellitus diagnosis, and insulin therapy

**adjusted for maternal BMI, maternal age, week of gestation of gestational diabetes mellitus diagnosis, insulin therapy, and fasting blood glucose concentration

OGTT, oral glucose tolerance test

Due to no statistically significant interaction between plasma glucose concentrations and the combined neonatal outcome, no analysis was conducted for this outcome.

Supplementary Table 5- Associations between OGTT results and adverse neonatal outcomes in women with GDM and SARS-CoV-2 infection, adjusted for COVID-19 related confounders

		Crude		Multivariable	
	Variable	OR [95% CI]	P	aOR [95% CI]*	P
Combined neonatal outcome	Fasting venous plasma glucose concentration [mmol/L]	1.03 [1.00;1.07]	**0.039**	1.04 [1.00;1.07]	**0.041**
	Venous plasma glucose concentration after 1 hr during OGTT [mmol/L]	1.00 [0.99;1.01]	0.520	1.00 [0.99;1.01]	0.575
	Venous plasma glucose concentration after 2 hrs during OGTT [mmol/L]	1.01 [0.99;1.02]	0.266	1.01 [1.00;1.02]	0.168
Premature birth	Fasting venous plasma glucose concentration [mmol/L]	1.06 [1.02;1.10]	**0.008**	1.05 [1.01;1.09]	**0.023**
	Venous plasma glucose concentration after 1 hr during OGTT [mmol/L]	1.01 [1.00;1.02]	0.135	1.01 [0.99;1.02]	0.118
	Venous plasma glucose concentration after 2 hrs during OGTT [mmol/L]	1.00 [0.99;1.02]	0.370	1.01 [0.99;1.02]	0.145

Data are presented as OR/aOR (95% CI) using logistic regression.

*adjusted for COVID-diagnosis after gestational diabetes mellitus diagnosis, variant type, vaccination status

OGTT, oral glucose tolerance test

Supplementary Table 6- Comparison of the final analytical sample of women with GDM and SARS-CoV-2 infection (CRONOS) and those who were excluded due to missing data

Characteristics	n	Excluded cases based on missing data	Final cohort	P
Maternal age	83/409	32 [29;36]	32 [28;36]	0.888
BMI	78/409	28.2 [23.9;32.7]	28.0 [24.2;33.1]	0.964
Vaccinated	79/383	24 (30.4%)	82 (21.4%)	0.084
Week of gestation of gestational diabetes mellitus diagnosis	120/409	25 [22;27]	26 [24;28]	**0.005**
Obesity	78/409	31 (39.7%)	159 (38.9%)	0.885
Insulin therapy	84/409	46 (54.8%)	148 (36.2%)	**0.002**

Data are presented as median [IQR] and as number/total number (percentage).

BMI, body mass index

Supplementary Table 7- Comparison of the final analytical sample of women with GDM and without SARS-CoV-2 infection (GestDiab) and those who were excluded due to missing data

Characteristics	n	Excluded cases based on missing data	Final cohort	P
Maternal age	2788/4598	32.0 [28.0;36.0]	33.0 [29.0;36.0]	**0.0001**
BMI		27.4 [23.7;32.1]	27.0 [23.3;32.0]	0.073
Week of gestation of gestational diabetes mellitus diagnosis		26.0 [25.0;28.0]	26.0 [25.0;28.0]	0.071
Obesity		976 (35.0%)	1553 (33.8%)	0.280
Insulin therapy		685 (24.6%)	1474 (32.1%)	**0.0001**
Data are presented as median [IQR] and as number/total number (percentage).<br>BMI, body mass index				
